# Emotional and cognitive experiences during the time of diagnosis and decision-making following a prenatal diagnosis: a qualitative study of males presented with congenital heart defect in the fetus carried by their pregnant partner

**DOI:** 10.1186/s12884-017-1607-y

**Published:** 2018-01-12

**Authors:** Tommy Carlsson, Elisabet Mattsson

**Affiliations:** 1 Department of Women’s and Children’s Health, Uppsala University, SE-75237 Uppsala, Sweden; 2 Department of Health Care Sciences, Ersta Sköndal University College, SE-10061 Stockholm, Sweden; 3 BMC, Husargatan 3, D11:1, SE-75237 Uppsala, Sweden

**Keywords:** Congenital heart defects, Fathers, Partners, Pregnancy, Prenatal diagnosis

## Abstract

**Background:**

Expectant fathers consider the second-trimester obstetric ultrasound examination as an important step towards parenthood, but are ill prepared for a detection of a fetal anomaly. Inductive research is scarce concerning their experiences and needs for support. Consequently, the aim of this study was to explore the emotional and cognitive experiences, during the time of diagnosis and decision-making, among males presented with congenital heart defect in the fetus carried by their pregnant partner.

**Methods:**

Twelve expectant fathers were consecutively recruited through two tertiary referral centers for fetal cardiology in Sweden, after they had been presented with a prenatal diagnosis of congenital heart defect in the fetus carried by their pregnant partner. The respondents were interviewed via telephone, and the interviews were analyzed using inductive qualitative content analysis.

**Results:**

The respondents experienced an intense emotional shock in connection with detection. However, they set their own needs aside to attend to the supportive needs of their pregnant partner, and stressed the importance of an informed joint decision regarding whether to continue or terminate the pregnancy. When terminating the pregnancy, they experienced a loss of a wanted child, an emotionally intense termination procedure, needs of support neglected by professionals, and worries about the risk of recurrence in future pregnancies. When continuing the pregnancy, they tried to keep a positive attitude about the coming birth, but were simultaneously worried about the postnatal situation.

**Conclusions:**

The findings illustrate the importance of inclusive care and adequate follow-up routines for both expectant parents following a prenatal diagnosis. This includes the initial emotional shock, the decisional process, and depending on decision reached, the termination or continuation of the pregnancy. Expectant fathers presented with a fetal anomaly need adequate follow-up routines to address worries about risk of recurrence in future pregnancies and worries about the postnatal situation.

## Background

Expecting a child is a life-transforming event [1], and expectant fathers go trough a process of adaptation to a parental role during the course of pregnancy, with increased emotional involvement as the pregnancy progresses [2–4]. This process of adaptation may be experienced as a stressful period, involving a number of psychological difficulties. The lack of tangible evidence of the pregnancy may make it feel unreal, resulting in attachment difficulties for expectant fathers. They experience a change in the relationship with their pregnant partner, with a discrepancy of the expectations and needs within the couple. Moreover, the situation requires a change in their core identity, with an adjustment to a new role as a parent, rather than a partner [4]. Although the needs of both expectant parents are acknowledged as equally important in healthcare, expectant fathers often feel excluded from the prenatal care, calling attention to the need for organizational changes and studies that address their specific experiences [5].

The obstetric ultrasound examination of pregnant persons in the second trimester of pregnancy, which involve the possibility to detect fetal anomalies, have been implemented as a part of maternity care in many countries around the world [6]. Expectant fathers consider the this examination an important step in their journey to become a parent [7], and desire to be involved in these pregnancy-related activities [5, 8, 9]. The examination is viewed by expectant parents as an opportunity to get a visual confirmation of the pregnancy, and considered an obvious choice because of the longing for the child [8]. However, many lack knowledge about the medical purposes of prenatal screening procedures [10, 11] and are thus often ill prepared for adverse findings, such as a prenatal diagnosis of a fetal anomaly [11–16].

Congenital heart defects are the most common of the fetal anomalies [17], and approximately one in five cases are diagnosed during pregnancy [17]. However, there is a great variation in the severity and prognosis depending on the nature of the defect [18, 19], and most of the severe defects are today prenatally diagnosed [17]. Research has shown that pregnant persons faced with a prenatal diagnosis of fetal anomaly experience a major life crisis [16, 20], involving a loss of the joy of being pregnant as well as the expected health of the fetus [16, 21]. Following the detection of a fetal anomaly, expectant parents may be presented with the option of terminating the pregnancy, a decision-making process that involves many informational and ethical considerations [22–27]. When told about the diagnosis, expectant parents move to a phase of trying to gain meaning and knowledge in order to reach an informed decision whether to continue or terminate the pregnancy [13]. However, an informed decision requires interpretation of complex medical information [28], and many persons who terminate the pregnancy experience the decision difficult to reach [22]. Expectant parents consider the decision to terminate the pregnancy to contradict deep parental instincts to protect one’s unborn child [29]. The decision entails a combination of chosen loss and lost choices, as the pregnancy is wanted while the fetal malformation is not [16]. Those faced with a prenatal diagnosis of fetal anomaly in the fetus carried by their pregnant partner show high levels of psychological distress and depression at the time of the diagnosis [30, 31]. When the pregnancy is terminated, a considerable proportion show symptoms of post-traumatic stress and depression, up to several months after the abortion [32, 33]. Expectant parents who continue the pregnancy show high levels of anxiety and depression during pregnancy [31], with expressions of intensive and varied positive as well as negative emotions [34, 35].

Research is scarce concerning the needs of support among expectant fathers [4] and their experiences when faced with a prenatal diagnosis of a fetal anomaly [20, 36]. Previous research has primarily focused on their role as supporters, rather than their own feelings and experiences [36]. Consequently, inductive qualitative research is needed to gain deeper insights into the experiences of these individuals. With this study, we wanted to address the scarcity of inductive research on the needs of care among this population, by giving attention to their own experiences in connection to the diagnosis and decision-making regarding whether to continue or terminate the pregnancy. Thus, the aim of this study was to explore the emotional and cognitive experiences, during the time of diagnosis and decision-making, among males presented with congenital heart defect in the fetus carried by their pregnant partner.

## Methods

### Study design

We conducted an inductive qualitative descriptive study, as we wanted to provide straight descriptions of the phenomenon and stay close to the collected material [37].

### Study context

In Sweden, midwives have primary responsibility for providing care to expectant parents with medically uncomplicated pregnancies and childbirths. The maternity care is a cost-free service that most expectant parents use. It consists of regular visits with the overall purpose to assess the status and progression of pregnancy, as well as to prepare expectant parents for childbirth and parenthood. At approximately 18 weeks of gestation, all expectant parents are offered a second-trimester obstetric ultrasound examination, with the medical purpose to estimate the gestational age, localize the placenta, assess the number of carried fetuses, and screen for fetal anomalies. When detected, suspected congenital heart defects are referred to a fetal cardiologist at a tertiary center for a specialist consultation. If a congenital heart defect is detected, the specialist offers information on a broad variety of topics related to the diagnosis, including the option of terminating the pregnancy. Before 18 completed weeks, pregnant persons may freely decide to terminate the pregnancy, and at later gestations approval for termination needs to be applied to the National Board of Health and Welfare. In clinical practice, pregnancy terminations are very seldom performed after 22 completed weeks [38].

### Sample

Respondents were consecutively recruited between March and November 2014 at two tertiary referral centers for fetal cardiology in central Sweden. To be eligible, participants needed to speak Swedish well enough to be interviewed and be presented with a prenatal diagnosis of major congenital heart defect in the fetus carried by their partner, diagnosed before 22 completed weeks of gestation. We define major congenital heart defect as in need of surgical repair within the child’s first year of life. During the inclusion period, 24 expectant fathers presented with a prenatal diagnosis of major congenital heart defect in the fetus were consulted at the centers. When approached face-to-face by the last author or a specialist nurse who worked at one of the sites, 20 consented to be contacted via telephone by the first author 5-15 weeks after the diagnosis. Four were not reached via telephone and four declined participation when contacted. Consequently, the final sample consisted of 12 respondents. Table 1 presents the characteristics of the sample. Among the terminated pregnancies (*n* = 7), four fetuses were diagnosed with univentricular defects, two with atrioventricular septal defects with associated trisomy 21, and one with pulmonary atresia/ventricular septal defect and major aortopulmonary collateral arteries. Among the continued pregnancies (*n* = 5), one fetus was diagnosed with a univentricular defect, three with tetralogy of fallot, and one with coarctation of the aorta. The fetuses were diagnosed between 17 and 20 weeks of gestation.

**Table 1:** Characteristics of the sample (*n* = 12)

Characteristic	Category	Terminated pregnancy (*n* = 7), n	Continued pregnancy (*n* = 5), n	Total (*n* = 12), n
Age	20-29	2	1	3
	30-39	3	3	6
	>39	2	1	3
Born children	0	2	2	4
	1	4	3	7
	2	1	0	1
Country of birth	Sweden	5	5	10
	Other	2	0	2
Highest education	Senior high school	3	2	5
	University/College	4	3	7

### Interviews

The respondents were interviewed via the telephone 5-15 weeks after the diagnosis (mean = 12 weeks). Semi-structured interviews were conducted by the first author, and lasted on average 35 min (range = 23-47) among those who continued the pregnancy and 37 min (range = 20-56) among those who terminated the pregnancy. Handwritten notes were kept during the course of the interviews, which were used to summarize it for the respondents. The interviews were digitally audio-recorded and transcribed verbatim. An interview guide was used to stay relevant to the aim (Table 2). Probes, such as “Can you please tell me more?”, were used to explore experiences further. At the end of the interview, the first author summarized his initial observations and asked if the participant wanted to correct or add anything. No repeat interviews were conducted.

**Table 2:** Interview guide

Main question	Sub-question
What was it like to be informed about the malformation?	What were your thoughts and feelings at that moment?
Could you describe how it was for you to make the decision to continue or terminate the pregnancy?	Was it difficult to make the decision?
	How would you describe your feelings and thoughts at the time?
	How did you feel when you were faced with the decision?
	Was there anything that was particularly difficult/easy?
Could you describe how you feel today?	What are your thoughts today about what happened?

### Analysis

The transcribed material was analyzed with inductive qualitative content analysis, a method to discover patterns in written texts [39] without preconceived theories or models to guide the analysis [40]. Nvivo version 10.2.0 (QSR International, Doncaster, Victoria, Australia) was used to structure the analytic process. The first author was responsible for the analysis. Initially, the transcripts were read several times to get a sense of the overall content. Meaning units were identified in the transcripts, defined as pieces of the text that represented a single item of content that corresponded to the aim of this study. The identified meaning units were condensed and then assigned a code, defined as a label that described the core of the meaning unit. The codes were abstracted to preliminary major themes, defined as overarching threads in the data that represent the underlying interpreted meaning. On account of the intertwined nature of human experiences, themes did not have to be mutually exclusive. Thus, codes could be placed in several themes [39]. Only the first author coded the transcripts. As more themes were identified, the last author was involved through discussions about the main findings and thematization. The analysis involved a continuous interpretive process that moved back and forth dialectically. For ethical reasons, no member checks took place. Table 3 presents examples of the analytic process.

**Table 3:** Examples of the analytic process

Meaning unit	Condensed meaning unit	Code	Theme
A heart defect is serious, of course, it really is, and I sound very calm about it but that is because there’s no reason not to believe it will go fine. That’s how we see it. Because if you don’t, you just can’t take it, I reckon	It is serious, and I sound very calm because there’s no reason not to believe it will go fine. That’s how we see it. If you don’t, you just can’t take it.	Expecting anything else than all will go well would not be possible to manage	Ambivalent feelings of anticipation and worries about the birth
That was really the thing that you pondered about the most, how it will, how will it feel to leave the child, what would happen if there’s chaos with the trains as there is every summer, things like that. What happens if we’re stranded, who has responsibility for us then.	That was the thing you pondered about most, how will it feel to leave the child, what happens if there’s chaos with the trains, things like that. What happens if we’re stranded, who has responsibility for us.	Pondering about the time after the birth	
It went like this, we had to deliver and that kind of thing, it became rather... emotional, still.	We had to deliver, it became rather emotional.	The pregnancy termination was emotional	The loss of a wanted child through an emotionally intense pregnancy termination
In part bad conscience I think. It feels like you terminate... someone else, so to say, you take away someone’s chance to live so to say.	In part bad conscience. Feels like you terminate... someone else, you take away someone’s chance to live.	Bad conscience to terminate a life	

### Research team and reflexivity

The authors are researchers, specialist nurses and midwives. The first author identifies himself as male and is in his 30’s, while the last author identifies herself as female and is in her 50’s. The first author had no contact with the respondents before the data collection. Before the interviews were initiated, the first author presented himself and stated the aim of the study. At the time of recruitment, the last author worked as a nurse-midwife at one of the units for fetal cardiology and was in charge of the recruitment from this site. The first author had no clinical experience of prenatal diagnostics before conducting the interviews. Both authors have previous experience of, and formal training in, interviews and qualitative content analysis.

## Results

The findings are portrayed through five themes: (1) Trying to support the partner during an emotional shock, (2) the importance of reaching an informed and joint decision, (3) the loss of a wanted child through an emotionally intense pregnancy termination, (4) ambivalent feelings of anticipation and worries about the birth, and (5) worries of recurrence in future pregnancies.

### Trying to support the partner during an emotional shock

The initial detection had been a complete emotional shock, described as a heartbreaking tragedy. When told about the suspected anomaly at the initial ultrasound examination, the respondents felt shocked, panicked, and disoriented. The wait between the initial detection and the consultation with the fetal cardiologist was described as very difficult, involving sleepless nights and many worries. When the fetal cardiologist confirmed the defect, respondents experienced the diagnosis surreal and unfair. While all reacted with shock to the initial detection, some distanced themselves from their own emotional reactions to cope with the situation. The expectant fathers mentioned the importance of supporting their pregnant partner, and because of this set their own needs aside to be attentive to the needs of their partners.

*I felt that I needed to be stable for my fiancée’s sake; depending on how she reacted I felt that I needed to be… more supportive than if I, if I broke down myself, if I put it like that...* (R6, pregnancy continued)

### The importance of reaching an informed and joint decision

The respondents emphasized the importance of being offered enough information in order to reach an informed decision whether to continue or terminate the pregnancy. Most considered they had reached the decision with certainty and did not regard any other option than the one they decided upon. Those who terminated the pregnancy perceived no other choice in order to protect their expected child from future suffering. On the contrary, respondents who continued the pregnancy regarded the severity of the heart defect as mild enough not to impose on the quality of life for the child and thus did not consider pregnancy termination.

*We would be happy to take over his pain ten-fold if that would make him pain-free. [...] It wasn’t particularly difficult bearing in mind that I don’t want to subject someone else to such pain.* (R10, pregnancy terminated)

*We haven’t really begun to think about if we should terminate the pregnancy, it doesn’t feel as if we have thought really hard along those lines actually, or so deeply about it, but it has always felt as if this will be OK in the end.* (R1, pregnancy continued)

The importance of reaching a joint decision together with their partner was brought up. It seemed that most couples came to a joint decision, and did not have any regrets over the final decision. However, some described not feeling included in the decision, which they in hindsight felt sad about.

*I wanted to go on but my girl preferred not to. […] I wanted to do everything to make her feel good, so I went along with most things. So it was more…her decision you might say in that respect. Unfortunately.* (R3, pregnancy terminated)

### The loss of a wanted child through an emotionally intense pregnancy termination

All respondents with experience of pregnancy termination described the situation as painful and emotionally stressful, involving complex ethical and existential issues. To terminate the pregnancy was described as a life-long burden, involving an alternative that no expectant parent should ever have to deliberate about. The expected child was deeply desired by respondents, who at the time of the diagnosis had started to look forward to becoming a parent. Thus, the alternative to terminate the pregnancy was regarded as a decision that partly contradicted their instincts to protect their unborn child. Simultaneously, they came to the conclusion that the decision was reached as a deed to spare the child from future postnatal suffering. Considering the existential aspects in play during the deliberation about the life-or-death options presented in connection to the diagnosis, the decision-making process was regarded as an inhumane situation, involving too great responsibilities for one person or couple to handle.

*I am playing God now. That is what I thought, playing God and deciding who was going to live and who was not going to live…* (R5, pregnancy terminated)

The pregnancy termination involved considerable emotional stress and the loss of a wanted child, likened by respondents to an execution and a murder. Some expressed negative experiences of abortion care. One referred to the pregnancy termination as a catastrophe, with unprofessional and stressed caregivers. Another drew attention to his vulnerable situation, and felt that the professionals had not been attentive to his needs.

*For something like this, the termination of a pregnancy, I have a bit of a feeling that they are not quite as good at taking care of the other partner. […] You feel a little bit like an appendage, to one side, but of course the partner is also in a helluva position.* (R8, pregnancy terminated)

Respondents who decided to view the fetus after the expulsion described it in both negative and positive terms. While an emotionally stressful moment, it also made the fetus feel more real, facilitated emotional recuperation, and brought closure. However, these respondents would have appreciated more time to deliberate about whether or not to view the fetus, as it was considered difficult to reach a decision when not presented with the option before the termination took place. Residual emotional scars and psychological consequences were experienced after the abortion, and thoughts about the loss of their expected child were often experienced among respondents. Moreover, they described feelings of guilt and shame over their decision, and pondered over how their situation would have been if the pregnancy had been continued.

*I’m still psychologically affected by it, naturally. I think most about the day when we had the abortion done.. It is that day in particular that comes back to my mind.* (R2, pregnancy terminated)

### Ambivalent feelings of anticipation and worries about the birth

Respondents with experience of a continued the pregnancy expressed ambivalent feelings about the future, as the birth of their child was anticipated and longed for, but accompanied by worries about the postnatal situation. Overall, the expectant fathers tried to keep a positive attitude towards the birth, as a way to cope with the situation. They were hopeful about the prognosis for the child, and relied on competent health care professionals to take care of their child once it was born.

*A heart defect is serious, of course, it really is, and I sound very calm about it but that is because there’s no reason not to believe it will go fine. That’s how we see it. Because if you don’t, you just can’t take it, I reckon […] It weighs you down a bit, of course, but... But... you have to see it as ... perfectly normal and not at all dangerous.* (R9, pregnancy continued)

However, many worries about the postnatal situation were also expressed. For example, these worries included the status of the child after birth, risks of planned surgeries, and managing the care of siblings during the critical postnatal periods. To deal with their worries and feel more in control, expectant fathers wished to be involved in the health care planning of the postnatal situation. One described his many thoughts and worries about taking care of the siblings after the child was born.

*It would just have been much easier if everything is ready here, like, you come down here as a family, stay here, we solve the problem... But it’s also like this, if there is room you can have your children and thoughts like that […] If you consider things you worry about, perhaps it’s in that area. How is he [the sibling] going to take it, like, will it be chaos, panic at home if we’re gone for a few days, as he’s so small.* (R1, pregnancy continued)

### Worries of recurrence in future pregnancies

Respondents with experience of a terminated pregnancy and one father whose child died shortly after birth described worries about the risk of recurrence in future pregnancies. These worries were very difficult to cope with, as new pregnancies were simultaneously longed for. As a way to deal with these worries, they expressed a need for expanded prenatal examinations in future pregnancies. To have experienced a prenatal detection of a fetal anomaly changed their views about obstetric ultrasound examinations, raising awareness of its medical purposes and influencing their feelings about the examination in future pregnancies.

*You’re a bit afraid that it, how can I put it, that next time, if there is a next time, that it will be another baby. You’ll, you can feel it even now, you’ll go and worry all the way up to the first ultrasound […] You feel that a bit even today, so to speak, a bit afraid that it will happen again.* (R10, pregnancy terminated)

## Discussion

The respondents experienced emotional distress in connection to the diagnosis and emphasized the importance of an informed joint decision. When the pregnancy was terminated, the procedure was described as emotionally painful, with health professionals not attentive to their needs for support. When the pregnancy was continued, respondents described worries related to the postnatal situation and wished to be involved in the planning of the postnatal care. Regardless of the decision to continue or terminate the pregnancy, respondents described that they tried to set their own needs aside to support their partner. According to previous studies, male partners faced with a prenatal detection of fetal anomaly hide their anxiety and grief in order to be strong for the pregnant person [12], resulting in concealment of their actual needs for support [41]. The results of this study confirm this, and bring to light that they, similar to pregnant persons, experience emotional distress following a prenatal diagnosis. The fact that expectant fathers put their feelings aside to be attentive to the supportive needs of their pregnant partner calls attention to the risk of overlooking their need for professional psychosocial support.

Previous studies report that women who terminate the pregnancy due to a fetal anomaly need compassionate care, but occasionally experience insensitivity and non-responsiveness from health professionals [15]. The findings of this study indicate that some male partners experience similar issues, and do not feel included in the abortion care. Moreover, the findings raise awareness of their vulnerable situation during pregnancy terminations, which is an incentive for health professionals to systematically assess and attend to the needs of both the pregnant person and their partner during this stressful process in their lives. A limited amount of research has been conducted concerning the psychological consequences of perinatal loss among male partners. The few reports that exist show that they grieve after a perinatal loss, although with less intensity and with other types of coping mechanisms than those of women [42]. Studies report that women suffer from psychological consequences years after a pregnancy termination due to a fetal anomaly [29, 32, 43]. This study provides an understanding about the emotional reactions among males faced with a prenatal diagnosis, namely the loss of a wanted child and worries about the risk of recurrence in future pregnancies. Considering the fact that we interviewed respondents a few weeks after the diagnosis, we cannot conclude the presence of complicated grief, defined as symptoms of grief that last at least 6 months [44]. However, continuity of care and adequate follow-up routines have previously been identified as important aspects of care for women terminating a pregnancy due to a fetal anomaly [45, 46]. The findings reflect that males need to be included in adequate follow-up routines, and illustrate the need for care that expands beyond the initial period following a pregnancy termination due to a fetal anomaly. To promote reproductive health and realistic expectations concerning the risk of recurrence, health professionals should take steps to address the thoughts and worries concerning future pregnancies. An example of an initiative that might promote reproductive health after a pregnancy termination due to a fetal anomaly is interconceptional counseling for couples, in other words care that focuses on the time between the termination and a new pregnancy or an informed decision not to conceive [47]. More research is needed to investigate appropriate timing of such counseling and its potential effects of promoting reproductive health among women and their partners after a pregnancy termination due to a fetal anomaly.

In line with other studies [21, 48, 49] expectant fathers with a continued pregnancy expressed ambivalent feelings about the birth of their child, suggesting the need for continuous care and support during the remainder of the pregnancy. Among expectant parents to prenatally diagnosed infants, gender differences may exist in the early postdiagnosis stage, with regard to psychological distress and quality of life. Nonetheless, both mothers and fathers show high levels in comparison to parents to healthy infants [31]. Moreover, 1 month after the disclosure, no gender differences exist with regard to emotional reactions [35, 50], and intracouple congruence of emotional reactions is frequent [50]. Consequently, health professionals must recognize that the diagnosis may result in an increased risk of developing psychopathological symptoms for both expectant parents when the pregnancy is continued, and include both of them in their assessments of the need for psychosocial support. Following the diagnosis, expectant parents move from grief to preparation as the pregnancy progresses [51] and are concerned or worried about various issues [21, 48]. In summary, the findings illustrate the importance of including both of the expectant parents in the health care planning of the postnatal situation, and the need for team-based efforts that address the worries of the postnatal situation expressed by the respondents.

### Strengths and limitations

The strength of this study is that it included males with experience of continuation and termination of pregnancy following a prenatal diagnosis of congenital heart defect in the fetus. Respondents with different ages, educational levels and fetal diagnoses were represented in the sample. Recruitment took place at two different tertiary units, indicating that the sample corresponds to different clinical settings. However, we did not use purposeful sampling to reach a sample of maximum variation [52], and half of the eligible persons declined participation or were not reached. We have no details regarding the characteristics of persons in the group who did not participate in the study. Consequently, it is possible that the interviewed respondents do not represent a varied sample from the complete population. For example, we failed to recruit any immigrants who continued the pregnancy. We acknowledge that the findings may have limited transferability to other settings and populations.

The material was collected using telephone interviews, a method that may promote respondents to feel more relaxed during the interview, increase anonymity, and lead to less influence from social pressure [53–56]. On the other hand, it may be more difficult to create a trusting atmosphere, use social cues [55], and it is impossible to observe nonverbal communication [56] via the telephone. During data collection, it became clear that the respondents appreciated the practical advantages of the chosen method. Further, we argue that it facilitated recruitment and helped participants to feel more relaxed when talking about the subject in question, which may have been a personal and intimate subject for some of the respondents. However, we cannot disregard the possibility that face-to-face-interviews may have generated richer data.

We acknowledge that this qualitative analysis is a product of using a single researcher as the primary instrument for analysis. To strengthen the credibility of the analysis, the first author kept a reflective journal about his preconceptions during the course of the data collection and analysis. Furthermore, the analytic process was discussed with the last author. This allowed for different perspectives and insights during the analytic process. Nevertheless, it is impossible to disregard the probability that our preconceptions had an impact on the analysis, and the findings needs to be interpreted with this in mind.

### Recommendations for future research

The findings implicate a need to longitudinally investigate the need for emotional support among expectant fathers faced with a prenatal diagnosis of fetal anomaly. Special attention needs to be directed towards couples that do not reach a joint decision regarding the future of the pregnancy. Research should also address worries of recurrence in future pregnancies and its potential impact on reproductive health. Moreover, research should explore how expectant parents that continue the pregnancy following the diagnosis most appropriately can be supported, so as to deal with their worries about the postnatal situation.

## Conclusions

This qualitative study adds insights into the existing literature, with regard to the emotional and cognitive experiences during the diagnosis and decision-making among males presented with congenital heart defect in the fetus carried by their pregnant partner. The findings illustrate the importance of inclusive care and adequate follow-up routines for both expectant parents following a prenatal diagnosis of congenital heart defect in the fetus. Despite experiencing an emotional shock in connection to the diagnosis, expectant fathers may put their own needs aside to support their pregnant partners. Consequently, they are at risk of not being adequately supported, and we suggest that professionals must always offer professional support to both expectant parents faced with a prenatal diagnosis of congenital heart defect in the fetus. This includes the initial emotional shock, the decisional process and, depending on decision reached, the termination or continuation of the pregnancy. Those with experience of pregnancy termination consider their decision as an option without alternatives, and describe the decision as an inhumane choice that contradicts their instincts to protect their unborn child. The experiences of insufficient support in connection to the termination calls attention to a need for abortion care that is inclusive, to adequately meet the emotional needs of males who experience a prenatal loss. Subsequent to the termination, these persons express worries of recurrence in future pregnancies, which needs to be addressed and followed up in order to promote realistic expectations about their reproductive futures. When the pregnancy is continued, partners express worries about the postnatal situation, raising awareness of the importance of continuous support during the remainder of the pregnancy. They need to be actively involved in the health care planning of the postnatal care, to feel prepared for the birth of the expected child.
